# Effect of impaired kidney function on outcomes and treatment effects of oral anticoagulant regimes in patients with atrial fibrillation in a real-world registry

**DOI:** 10.1371/journal.pone.0310838

**Published:** 2024-09-23

**Authors:** Christian Salbach, Barbara Ruth Milles, Hauke Hund, Moritz Biener, Matthias Mueller-Hennessen, Norbert Frey, Hugo Katus, Evangelos Giannitsis, Mustafa Yildirim

**Affiliations:** Department of Internal Medicine III, Cardiology, University Hospital of Heidelberg, Heidelberg, Germany; Sapienza University of Rome: Universita degli Studi di Roma La Sapienza, ITALY

## Abstract

**Background:**

The impact of impaired kidney function on outcomes and treatment benefits of vitamin-K antagonists (VKA) versus direct oral anticoagulants (DOAC) in patients with atrial fibrillation (AF) has insufficiently been investigated in randomized controlled studies (RCTs). Most studies and registries are either biased due to incomplete enrolment of consecutive patients in large pharma industry sponsored registries, or due to short recruitment periods or incomplete assessment of important variables in national registries.

**Methods:**

This study uses data from the Heidelberg Registry of Atrial Fibrillation (HERA-FIB), a retrospective single-center registry of 10,222 consecutive patients with AF presenting to the emergency department of University Hospital of Heidelberg from June 2009 until March 2020. Rates of all-cause mortality, stroke, major bleeding and myocardial infarction (MI) were related to the presence and severity of impaired presenting kidney function, as well as to assigned treatment with VKA vs. DOAC.

**Results:**

The risks for all-cause mortality (HR: 3.26, p<0.001), stroke (HR: 1.58, p<0.001), major bleeding (HR: 2.28, p<0.001) and MI (HR: 2.48, p<0.001) were significantly higher in patients with an eGFR<60 ml/min at admission and increased with decreasing eGFR. After adjustment for variables of CHA_2_DS_2_VASc-score, presence of eGFR <60 ml/min remained as an independent predictor for all-cause mortality, major bleeding and MI. The hazard ratio (HR) for all-cause mortality, major bleedings and MI was significantly lower in patients receiving DOAC compared to VKA.

**Conclusion:**

Findings from our large real-life registry confirm the data from RCTs and extend our knowledge on the effectiveness and safety of DOACs to subjects that were underrepresented in RCTs.

## Introduction

In the setting of an emergency department (ED), patients with atrial fibrillation (AF) and impaired kidney function are frequently encountered [1, 2]. Additionally, the presence of chronic kidney disease (CKD) has also been reported to independently increase the incidence of AF [1]. In line with the demographic trend of an aging population, the incidence of patients suffering from both AF and CKD is growing [1–3]. Herein, evidence suggests a higher risk of stroke, myocardial infarction (MI) and major bleedings for patients with CKD [2, 4, 5]. Although, CKD adds prognostic information to the CHA_2_DS_2_VASc-score, rates on the implementation of the new R_2_CHA_2_DS_2_-VASc score [6] which is modified by adding the kidney disease component to the traditional CHA_2_DS_2_VASc-score are presumably low in clinical routine. Based on the major randomized controlled trials (RCTs), current guidelines on AF recommend an initiation of an oral anticoagulant (OAC) therapy in AF patients, favoring direct oral anticoagulants (DOAC) accounting to the lower risk of major bleedings [7]. However, specific populations, such as those with severely impaired kidney function are still underrepresented in RCTs and only few registries on AF provide information on unselected all-comers recruited over a long time period, leaving a gap of evidence for patients with AF and reduced kidney function in clinical practice [5, 8]. Therefore, this study aimed to provide data on the prevalence of reduced kidney function and severity in AF patients presenting to an ED, stratified for outcome data encompassing all-cause mortality, stroke, major bleedings and MI. Additionally, we aimed to investigate the impact of DOAC compared to vitamin-K antagonist (VKA) in AF patients with reduced kidney function in a large monocentric registry on all-comers to an emergency department (ED).

## Materials and methods

### Study population and design

The study uses data from the Heidelberg Registry of Atrial fibrillation (HERA-FIB). Data collection and study population of this retrospective single-center study was described earlier [9]. Included patients were consecutively admitted to the ED of the University Hospital of Heidelberg from June 2009 to March 2020 with AF. Inclusion and exclusion criteria for HERA-FIB were already described [9]. HERA-FIB includes patients ≥18 years with the diagnosis of AF, either as primary reason or as a comorbidity with at least one available high sensitive cardiac troponin T (hsc-TnT) laboratory value at admission. Additionally, patients without complete follow-up on all-cause mortality were excluded [9]. This retrospective observational study had no influence on patient treatment. All treatments were provided at the discretion of the treating physicians. Data was accessed for research purposes 1^st^ April 2024. Except for outcome variables, all variables were captured at index admission to the ED. The authors had no access to information that could identify individual participants after data collection. This study was approved by the local ethics committee of the Medical Faculty of Heidelberg. Informed consent for this retrospective analysis was waived by local ethics committee. This study was conducted according to ethical principles stated in the Declaration of Helsinki. Patient identifiable data was pseudonymized to ensure data confidentiality and was not passed on to third parties. This study is registered at ClinicalTrials.gov. ClinicalTrials.gov identifier: NCT05995561.

### Follow-up and data

A sequential follow-up was performed as described [9]. Herein, first, data from the hospital information system and hospital files from other hospitals affiliated with the University Hospital of Heidelberg were screened for information on outcome variables. Afterwards, structured patient phone calls were executed; if not possible, postal queries with standardized questionnaires were conveyed. If patients were still unattainable, registration offices were contacted, which could provide data on vital status and residency. If there was no information on outcome parameters such as stroke, major bleeding or MI, patients were excluded in analyses regarding these endpoints. A composite endpoint for all-cause mortality, ischemic stroke, major bleeding and MI was assessed [9]. Analyses were performed after removing and exclusion of missing data. For the composite endpoint, patients were censored on the first event. The data that support the findings of this study are available from the corresponding author upon reasonable request.

### Definitions

Reduced kidney function at admission was diagnosed if the estimated glomerular filtration rate (eGFR) was < 60 mL/min per 1.73 m^2^, using the CKD-EPI (Chronic Kidney Disease Epidemiology Collaboration) formula. Since urine albumin or other markers of kidney injury were not collected systematically, additional components of impaired kidney function were not considered [10]. The CHA_2_DS_2_VASc-score was calculated retrospectively for all patients by adding two points for age ≥ 75 years and prior stroke, transient ischemic attack (TIA) or thromboembolism, as well as one point for congestive heart failure (defined by left ventricular ejection fraction <50%), arterial hypertension, age between 65–74 years, known diabetes mellitus, prior MI or peripheral artery disease, and female gender, respectively [7, 11, 12]. We also calculated the HASBLED [13] and ORBIT score [14] for evaluating bleeding risk. HASBLED score was calculated for all patients as described [13] by adding one point for arterial hypertension, abnormal liver function (defined as chronic hepatic disease e.g. cirrhosis), abnormal renal function (defined as creatinine >2.26 mg/dl), history of stroke or history of bleeding, labile INR, age > 65 years, and usage of nonsteroidal or anti-inflammatory drugs, as well as co-medication of aspirin or clopidogrel. ORBIT score was calculated as defined [14] by adding 2 points for hemoglobin levels <13 g/dL for males and <12 g/dL for females, 1 point for age > 74 years, 2 points for history of bleeding, 1 point for an eGFR< 60 ml/min/1.73m^2^ and 1 point for treatment with antiplatelet agents. A HASBLED score ≥3 and an ORBIT score ≥4 were regarded as high bleeding risk. A major bleeding event was defined according to the International Society on Thrombosis and Hemostasis (ISTH) major bleeding criteria [11]. Stroke definition excluded hemorrhagic stroke, which was allocated as a major bleeding event according to the ISTH major bleeding classification but included ischemic or unknown causes of stroke. A myocardial infarction was defined using the universal MI definition [15].

### Statistical analysis

Continuous variables were tested for normal distribution using the Kolmogorov-Smirnov test. Parametric data is presented as means (standard deviations, SD), non-parametric data as medians (25th, 75th percentiles, IQR). Groups were compared using chi-squared test or Fisher’s exact test for categorical variables, and unpaired Student’s t-test or Wilcoxon rank-sum test for continuous variables. For Kaplan-Meier analyses the log rank test was used. A multivariate Cox proportional hazards model was used to determine independent predictors for all-cause mortality, stroke, major bleedings and MI. The proportional hazards assumption was tested using the Grambsch and Therneau method. Time-dependent receiver-operating-characteristic (ROC) curves from censored survival data using the Kaplan-Meier method were estimated and the area under the ROC curves (AUC) was calculated. The 95% confidence interval (CI) of AUC was calculated according to Hanley and McNeil. A two tailed P-value of <0.05 was considered to indicate statistical significance. Statistical analyses were performed using MedCalc Version 20.105. R software (version 4.3.0, R Foundation for Statistical Computing, Vienna, Austria) was utilized for calculating the Net Reclassification Index (NRI).

## Results

The HERA-FIB cohort consisted of a total of 10,222 patients. Among these, 6,334 (62%) presented with an eGFR ≥60 ml/min and 3888 (38%) with an eGFR <60 ml/min. A total of 2,988 (29.2%) patients presented with an eGFR between 30–59 ml/min, 653 (6.4%) with an eGFR between 15–29 ml/min and 247 (2.4%) as with an eGFR <15 ml/min. An eGFR ≤50 ml/min was present in 2,734 (26.7%)patients. Baseline characteristics are reported for subgroups based on the presence or absence of an eGFR <60 ml/min (**Table 1**). The groups differed significantly concerning the baseline variables. The majority of patients with reduced presenting kidney function was male 2,088 (53.7%). Median age was higher in patients with eGFR <60 ml/min. Cardiovascular risk factors, comorbidities, the CHA_2_DS_2_VASc-score and biomarkers such as hs-cTnT were significantly higher in patients with an eGFR <60 ml/min. The median HASBLED- and ORBIT-score also differed classified by eGFR <60 ml/min.

**Table 1:** Baseline characteristics classified by presence or absence of eGFR >60 ml/min.

Variables	eGFR ≥60 ml/min.	eGFR <60 ml/min.	p-value
Age, y, median (IQR)	72 (62–79)	79 (73–85)	<0.001
Sex, male, n (%_all_)	3869 (61.1)	2088 (53.7)	<0.001
BMI, kg/m^2^, median (IQR)	26.7 (24.0–30.5) n = 2538	26.8 (24.1–30.7) n = 3844	0.32
HR, bpm, median (IQR)	94 (75–122)	85 (70–110)	<0.001
Bp_syst_, median (IQR)	148 (134–161) n = 6295	144 (126–159) n = 3869	<0.001
Bp_diast_, median (IQR)	88 (78–99) n = 6293	80 (70–92) n = 3868	<0.001
Arterial hypertension, n (%_all_)	4913 (77.6)	3523 (90.6)	<0.001
Diabetes mellitus, n (%_all_)	1002 (15.8)	1041 (26.8)	<0.001
Former CAD, n (%_all_)	2360 (37.3)	2054 (52.8)	<0.001
Former CABG, n (%_all_)	434 (6.9)	508 (13.1)	<0.001
Former MI, n (%_all_)	840 (13.3)	832 (21.4)	<0.001
Former COPD, n (%_all_)	588 (9.3)	506 (13.0)	<0.001
CHA_2_DS_2_VASc-score*, median (IQR)	3 (2–5)	5 (4–6)	<0.001
HASBLED-score*, median (IQR)	2 (1–3)	2 (2–3)	<0.001
ORBIT-score*, median (IQR)	1 (0–2)	3 (2–4)	<0.001
Hs-cTnT, ng/L, median (IQR)	13 (8–23)	34 (19–64)	<0.001
Serum creatinine, mg/dl, median (IQR)	0.85 (0.7–1.0)	1.43 (1.2–1.8)	<0.001
eGFR^#^, ml/min., median (IQR)	81.8 (71.2–92.1)	42.4 (31.0–51.6)	<0.001
OAC, n (%_all_)	4520 (71.3)	2606 (67.1)	<0.001
DOAC, n (%_all_)	2922 (46.1)	1319 (33.9)	<0.001
VKA, n (%_all_)	1598 (25.2)	1287 (33.1)	<0.001
s.c. anticoagulant**, n (%_all_)	625 (9.8)	433 (11.1)	0.0384
No anticoagulation, n (%_all_)	1189 (18.8)	849 (21.8)	<0.001

*individual components of the respective scores are shown in **S1 Table**

^#^eGFR was calculated using CKD-EPI formula

** s.c. anticoagulant included regimes with Heparin, LMW-Heparin or Fondaparinux;BMI, body mass index; AF, atrial fibrillation; HR heart rate; bpm, beats per minutes; bp blood pressure; sys, systolic; dia, diastolic; CAD, coronary artery disease; CABG, coronary artery bypass graft surgery; MI, myocardial infarction; hs-cTnT, high sensitive troponin T, NTproBNP, n-terminal-pro brain natriuretic peptide; eGFR, estimated glomerular filtration rate; OAC, oral anticoagulation; DOAC, direct oral anticoagulant; VKA, vitamin K antagonist; s.c. subcutaneous.

### Outcomes stratified by eGFR

**Fig 1:**
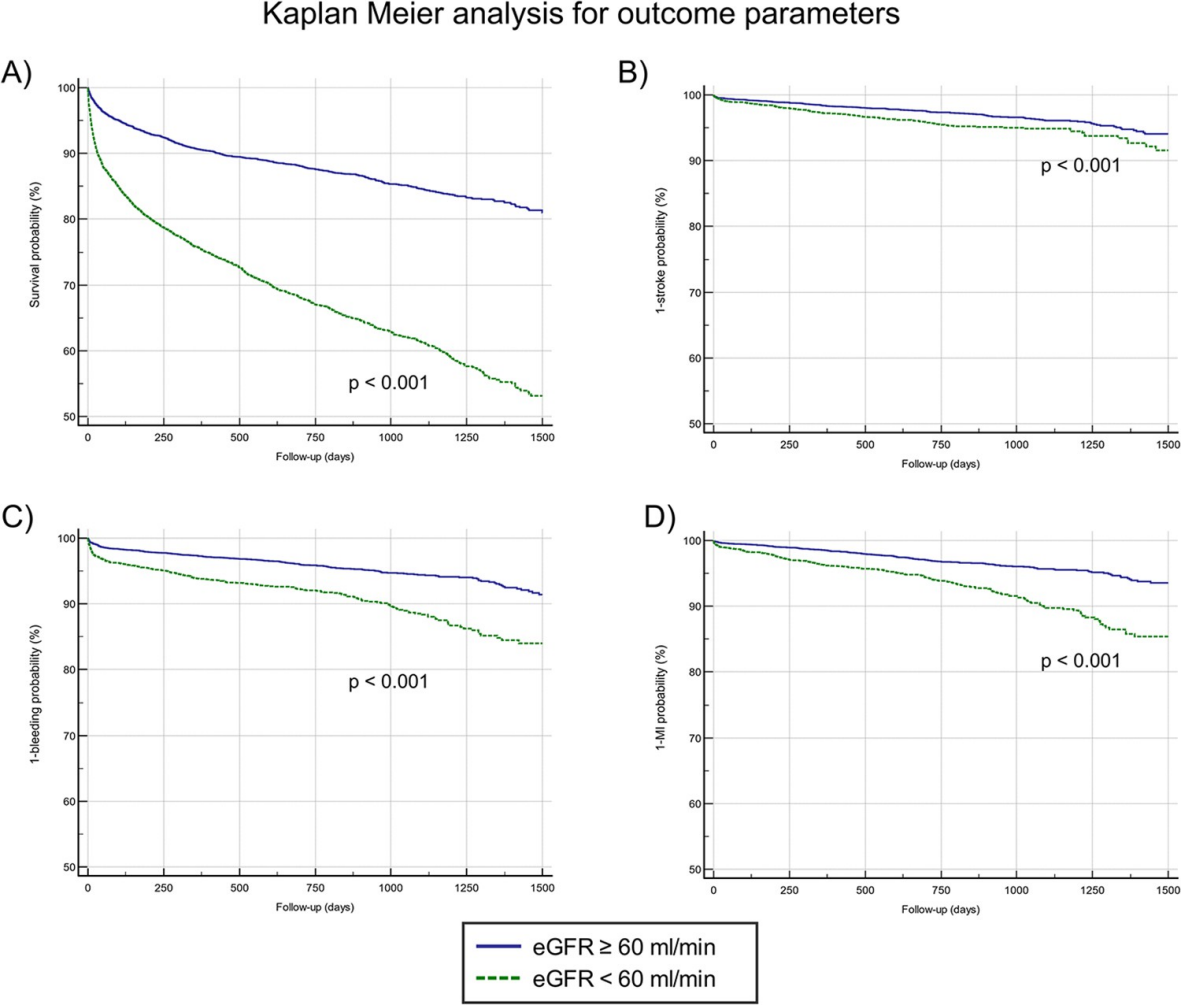
Kaplan-Meier analysis showing probability for all-cause mortality, stroke, major bleeding and myocardial infarction classified by presence or absence of eGFR <60 ml/min. Patients with a reduced eGFR at admission showed a higher all-cause mortality (A), a higher probability of stroke (B), a higher probability of major bleeding (C) and a higher probability for myocardial infarction (D).eGFR, estimated glomerular filtration rate, MI; myocardial infarction.

Outcomes and endpoints categorized by the presence or absence of eGFR <60 ml/min are shown in **Table 2**. During a median follow-up of 23 months (IQR 12–35), a total of 2,173 (21.3%) patients died, 1,325 (34.0%) with and 848 (13.4%) without eGFR <60 ml/min. 287 experienced a stroke, 122 (3.8%) with and 165 (3.0%) without eGFR <60 ml/min., 514 (5.9%) suffered a major bleeding event whereas 261 (8.2%) with and 253 (4.6%) without eGFR <60 ml/min. and 382 (3.7%) a MI 198 (6.2%) with and 184 (3.3%) without eGFR <60 ml/min. Kaplan-Meier analysis for all-cause mortality, stroke, major bleeding and MI categorized by the presence or absence of a reduced presenting kidney function is shown in **Fig 1**. Here, log rank tests were significant for all-cause mortality (HR: 3.27 95%CI: 2.99–3.57, p<0.001), stroke (HR: 1.58 95%CI: 1.23–2.02, p<0.001), major bleeding (2.28 95%CI: 1.90–2.74, p<0.001), MI (2.48, 95%CI: 2.00–3.10, p<0.001), as well as the composite endpoint (2.68 95%CI: 2.49–2.90, p<0.001). A cox regression analysis for all-cause mortality revealed age, male sex, diabetes mellitus, former CAD, CABG and COPD, a hs-cTnT ≥14 ng/L as well as a reduced presenting kidney function < 60 ml/min to be independently associated with all-cause mortality (**S2 Table**).

**Table 2:** Outcomes classified by presence or absence of eGFR <60 ml/min.

Variables	eGFR ≥60 ml/min	eGFR <60 ml/min	p-value
All-cause mortality, n (%_all_)	848 (13.4)	1325 (34.0)	<0.001
Stroke, n (%_all_)	165 (3.0) n = 5528	122 (3.8) n = 3194	0.03
Major bleeding, n (%_all_)	253 (4.6) n = 5528	261 (8.2) n = 3197	<0.001
Myocardial infarction, n (%_all_)	184 (3.3) n = 5516	198 (6.2) n = 3188	<0.001
Composite endpoint*, n (%_all_)	1271 (20.1)	1642 (42.2)	<0.001

*The composite endpoint included all-cause mortality, stroke, major bleeding and myocardial infarction. eGFR; estimated glomerular filtration rate.

### Modification of the CHA_2_DS_2_VASc-score

In a multivariate Cox proportional hazards model, presence of eGFR <60 ml/min, as well as variables that compose the CHA_2_DS_2_VASc-score were included. Here, presence of a reduced kidney function emerged as an independent predictor for all-cause mortality (aHR 2.18 95% CI: 2.00–2.40, p<0.001), major bleedings (aHR 1.68 95% CI: 1.40–2.03, p<0.001) and MI (aHR: 1.59 95%CI: 1.29–1.97, p<0.001), however not for stroke (aHR: 1.08 95%CI: 0.84–1.38 p = 0.55). For prediction of the composite endpoint consisting of all-cause mortality, stroke, major bleedings and MI, an eGFR <60 ml/min was independently associated with an adverse outcome (aHR: 1.85 95%CI 1.71–2.00; p<0.001). Respective Cox proportional hazard models are shown in **S3–S7 Tables**. The addition of eGFR <60 ml/min with 2 points as described within the calculation of the R_2_CHA_2_DS_2_VASc-score [6] significantly improved the performance for the prediction of all-cause mortality and major bleedings compared to the original CHA_2_DS_2_VASc-score as indicated by an increase of the AUC from 0.642 (95%CI: 0.632–0.651) to 0.684 (95%CI 0.674–0.693), Δ-AUC 0.0421, p<0.001, and from an increase of AUC: 0.603 (0.593–0.613) to 0.619 (95%CI: 0.609–0.629), p = 0.01, respectively. However, concerning the performance of prediction for stroke and MI the addition of eGFR <60 ml/min did not improve the original CHA_2_DS_2_VASc-score, AUC: 0.623 (95%CI: 0.612–0.633) vs. 0.614 (95%CI: 0.604–0.624,) p = 0.2708, and AUC: 0.632 (95%CI 0.622–0.642) vs. 0.641 (95%CI: 0.631–0.651), p = 0.2239, respectively. Certainly, the addition of eGFR <60 ml/min improved the performance of the CHA_2_DS_2_VASc-score for the prediction of the composite endpoint from an AUC of 0.645 (95%CI: 0.635–0.654) to 0.677 (95%CI: 0.667–0.686), p<0.001. The continuous NRI for the addition of eGFR <60 ml/min to the CHA_2_DS_2_VASc-score for all-cause mortality was 0.278 (95%CI 0.251–0.300), p<0.001. For major bleedings, continuous NRI was 0.222(95% CI: 0.130–0.269), p<0.001.

### Bleeding risk in patients with an eGFR <60 ml/min

For comparison of bleeding risk scores, HASBLED- and ORBIT-score were calculated. Using a comparison between AUCs, ORBIT score showed a better prediction of a major bleeding event in all patients compared to HASBLED score AUC: 0.603 (95%CI: 0.593–0.613) vs. 0.660 (95%CI: 0.650–0.670), p<0.001. The continuous NRI also showed a better prediction of a major bleeding event for ORBIT- compared to HASBLED-score and was 0.242 (95% CI: 0.173–0.348), p<0.001. Therefore, we compared the performance of HASBLED and ORBIT score in patients with and without eGFR<60 ml/min. For patients with eGFR <60 ml/min at presentation, AUCs of both scores showed a weak performance, but the HASBLED score differed significantly compared to the ORBIT score: 0.540 (95%CI: 0.523–0.558) vs. 0.587 (95%CI: 0.570–0.604), p = 0.0188. When considering patients with an eGFR >60 ml/min, the ORBIT score showed a better performance compared to the HASBLED score: 0.627 (95%CI: 0.614–0.640) vs. 0.667 (95%CI: 0.654–0.679), p = 0.0290. This was also confirmed by the continuous NRI with 0.189 (95% CI: 0.0–0.322), p<0.001, favorizing the ORBIT-score for prediction of a major bleeding event. Since current evidence suggests a non-difference of discrimination of HASBLED and ORBIT score in patients receiving OAC for bleeding, we sought to compare HASBLED and ORBIT score for prediction of a major bleeding event in AF patients receiving DOAC regimes. Here, we were able to show a better performance of ORBIT score compared to HASBLED score: AUC 0.591 (95% CI: 0.578–0.603) vs. 0.648 (0.636–0.660), p = 0.0005 for predicting a major bleeding event in AF patients on DOAC regimes. This superiority was also confirmed by a continuous NRI of 0.211 (95% CI: 0.01–0.357), p<0.05, favoring the ORBIT score.

### Effect of a reduced eGFR on outcomes

**Fig 2:**
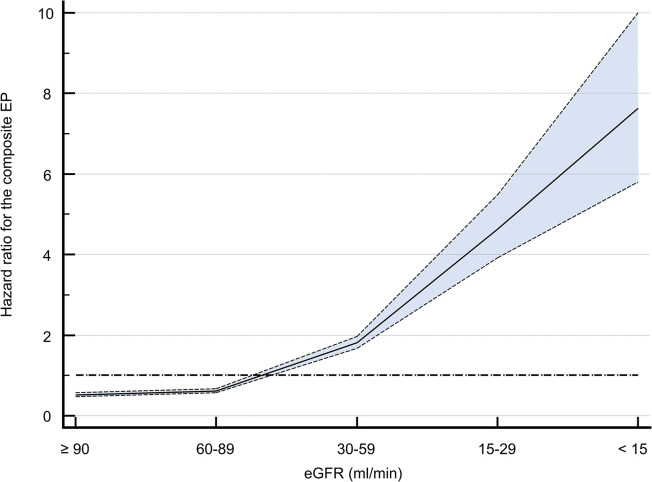
Rates for the composite EP consisted of all-cause mortality, stroke, major bleedings and MI increased as eGFR declined. eGFR, estimated glomerular filtration rate; EP, endpoint.

HR for the composite EP consisting of all-cause mortality, stroke, major bleedings and MI increased as eGFR declined (**Fig 2**). Here, HRs for all-cause mortality, stroke, major bleedings and MI in patients with a CKD stage of at least stage 4 or 5 were 7.19 (95%CI: 6.10–8.47, p<0.001) for all-cause mortality, 2.24 (95%CI: 1.39–3.60, p = 0.0009) for stroke, 6.63 (95%CI: 4.66–9.42, p<0.001) for major bleeding, 3.53 (95%CI: 2.33–5.35, p<0.001) for MI and 5.22 (95%CI: 4.52–6.02, p<0.001) for the composite endpoint. Kaplan Meier analysis separated by eGFR stages is shown in the supplement (**S1 Fig**). Even after adjustment for significant univariate variables, aHR for the composite EP increases, as eGFR declined (aHR_eGFR:60–89 ml/min_ 0.93 (95%CI: 0.81–1.07), p = 0.3337, aHR_eGFR:30–59 ml/min_ 1.17 (95%CI: 1.1.03–1.37), p = 0.0207, aHR_eGFR:15–29 ml/min_ 1.71 (95%CI: 1.44–2.02), p<0.0001), aHR_eGFR:<15 ml/min_ 1.87 (95%CI: 1.52–2.27), p<0.0001. A cox regression model for eGFR stages and respective p of interactions are shown in **S8 Table**. Herein, age and former COPD showed a significant interaction with the composite EP and eGFR stages.

### Role of VKA and DOAC through the recruitment period

**Fig 3:**
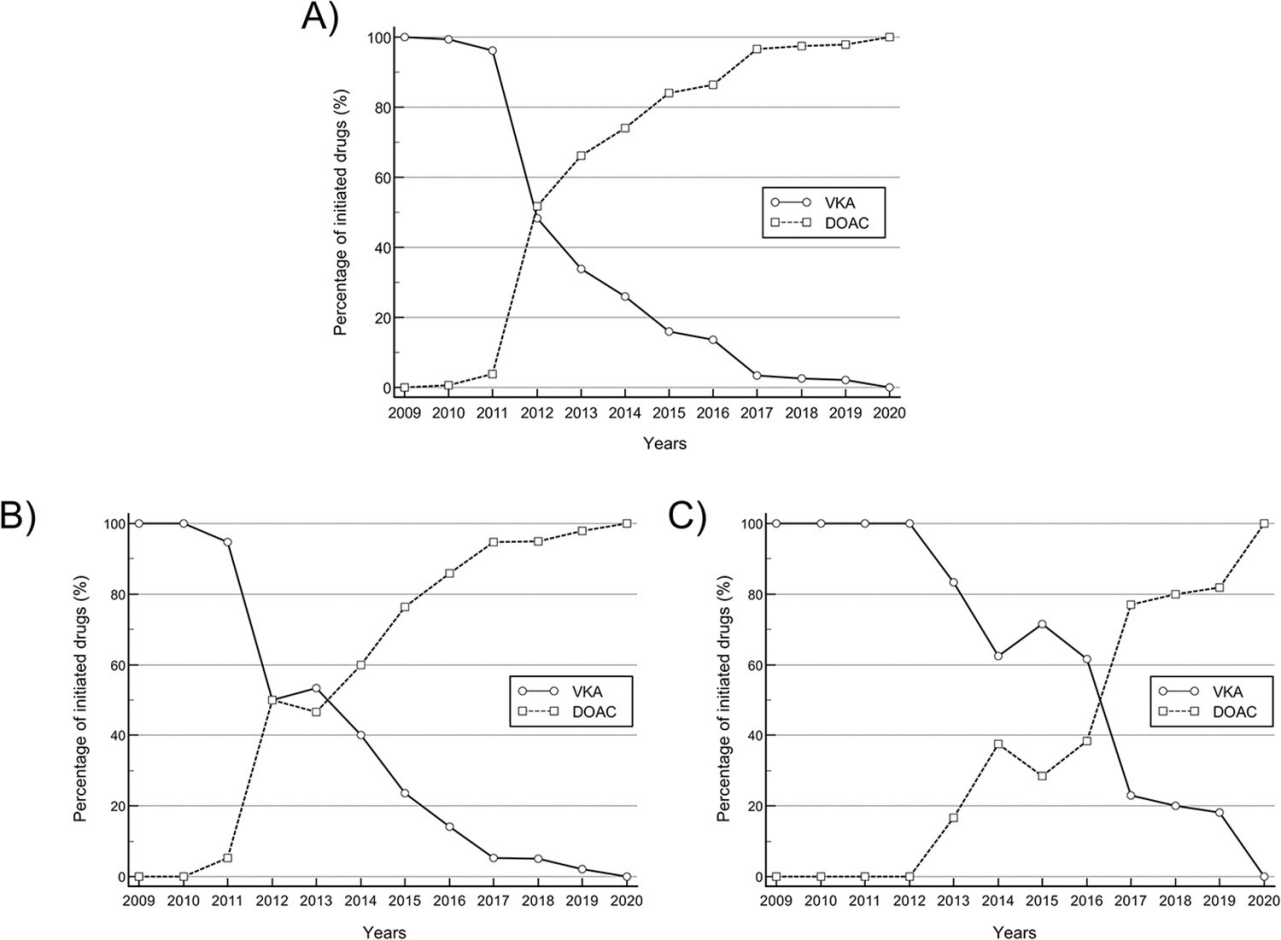
Comparison of initiation rates with oral anticoagulation regimes containing DOAC and VKA. Initiation rates of VKA decline as initiation rates for DOAC regimes increased (A). Initiation rates of DOAC in patients with an eGFR 30–59 ml/min. (B) was earlier compared to patients with an eGFR <30 ml/min. (C). DOAC, direct oral anticoagulant; VKA, vitamin-K antagonist.

Numbers of subjects with new initiation of DOAC increased during the entire recruitment period from with a concomitant decrease of VKA initiations (**Fig 3A**). **Fig 3B** shows initiation rates of DOAC, and VKA in patients with eGFR 30–59 ml/min and **Fig 3C** for patients with eGFR <30 ml/min, respectively. Here, the initiation of OAC regimes consisting of a DOAC increased throughout the recruitment period. In total, regimens containing DOAC in patients with an eGFR 30–59 ml/min were initiated earlier compared to patients with an eGFR <30 ml/min. The distribution of patients reciving a DOAC classified by DOAC type and eGFR category is shown in **S2 Fig**.

### Effect of OAC regimens on outcomes

**Fig 4:**
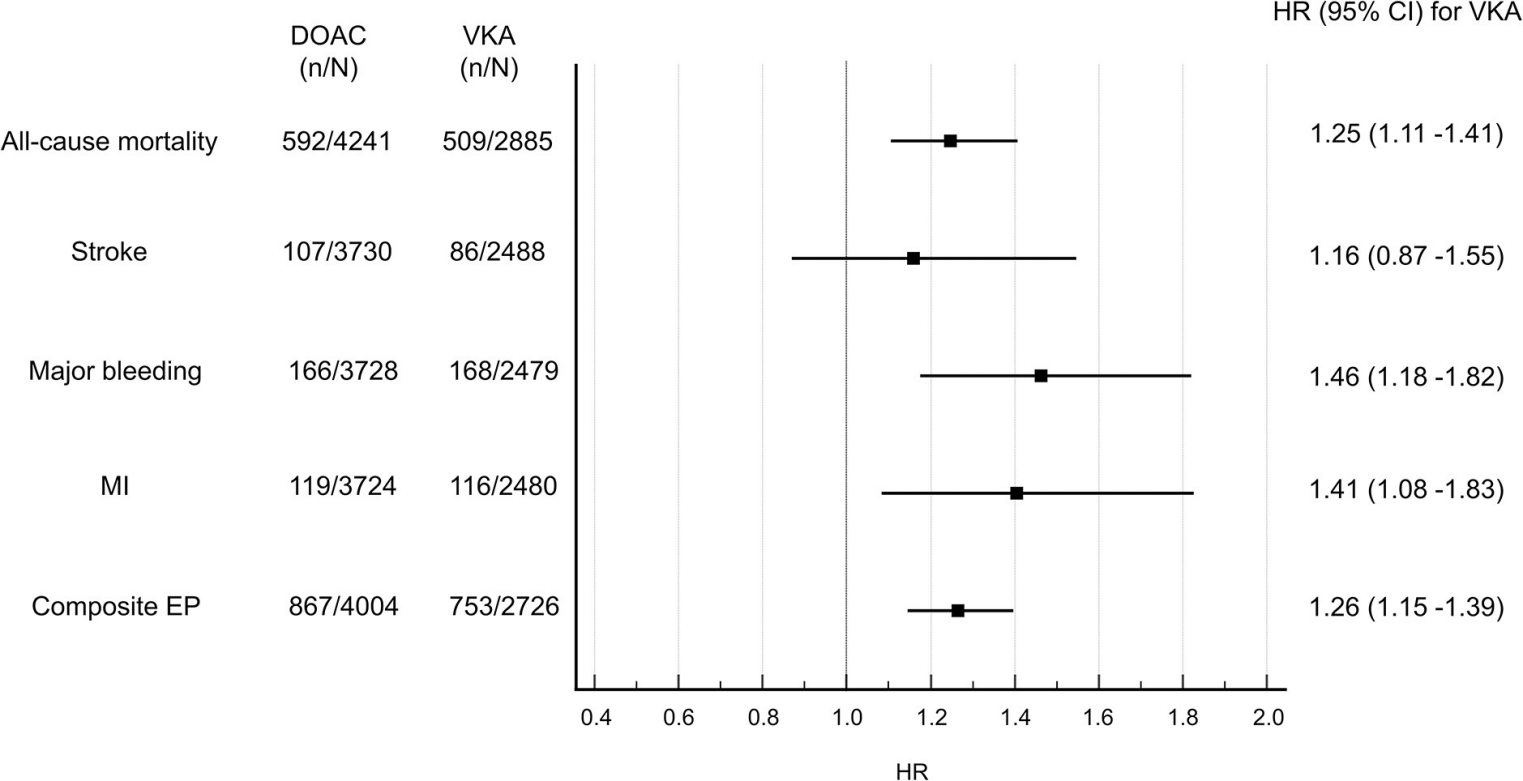
Forrest plot for HRs and outcome parameters classified by OAC regimes containing VKA or DOAC. HR, hazard ratio; CI: confidence interval; VKA, vitamin-K antagonist; DOAC, direct oral anticoagulant, EP; endpoint.

A forest plot and related hazard ratios (HRs) with respective 95% CI classified by outcome parameters (all-cause mortality, stroke, major bleedings, MI and the composite endpoint) according to OAC regimes (VKA vs. DOAC) for AF patients receiving an OAC is shown in **Fig 4**. Except for stroke, where no significant differences could be detected in patients receiving DOAC vs. VKA (HRs: 0.86 95%CI: 0.65–1.15 vs. 1.16 95% CI 0.87–1.55, p = 0.31), for all other outcome parameters, such as all-cause mortality (HR: 0.80 95%CI: 0.71–0.91 vs. 1.25 95%CI 1.11–1.41, p<0.001), major bleeding events (HR: 0.68 95%CI 0.55–0.85 vs. 1.46 95%CI 1.18–1.82, p<0.001), MI (HR: 0.71 95%CI 0.55–0.92 vs. 1.41 95%CI 1.08–1.83 p = 0.01), as well as the composite endpoint (HR: 0.79 95%CI 0.72–0.87 vs. 1.26 95%CI 1.15–1.39, p<0.001), HRs for respective outcomes in AF patients receiving a DOAC regime were significantly lower versus patients receiving a VKA.

## Discussion

In this study, we report several interesting findings. First, we report a high prevalence of a reduced kidney function at admission indicated by an eGFR <60 ml/min which was 38%. Second, patients with an eGFR <60 ml/min at admission showed higher risks for all-cause mortality, major bleedings and incident MI. Third, we report an eGFR <60 ml/min at admission to be an independent predictor for all-cause mortality, major bleedings and incident MI even after adjustment for components of the CHA_2_DS_2_VASc-score. Fourth, the HR for all-cause mortality, major bleedings and MI was significantly lower in patients receiving DOAC compared to patients receiving a VKA.

In a meta-analysis of DOAC phase III RCTs in patients with AF by Ruff et al., the prevalence of CKD, defined as creatinine clearance (CrCl) ≤50 ml/min, was 13% (1134 of 8722 subjects) [16]. However, current RCTs excluded patients with CrCl <25ml/min [17] and <30 ml/min [18–20]. Thus, the prevalence of AF can be substantially higher in CKD populations. In the CRIC (Chronic Renal Insufficiency Cohort) study, the prevalence of non-valvular AF was 18% [21]. The pooled prevalence of AF or atrial flutter was 16.5% based on measured CKD in a cohort of 2.4 million CKD patients with data retrieved from digital healthcare data from 11 countries [22]. In the 2018 annual US renal data report, AF prevalence increases as CKD progresses, ranging from 21.3% in stages 1–2 to 28.3% in stages 4–5 [23]. Hence, the prevalence of a reduced presenting kidney function, defined as eGFR <60 ml/min/1.73m^2^ in our AF all-comer registry was 38%. Here, we report a higher prevalence of eGFR <60 ml/min among AF patients compared to previous reports, potentially owing to the consecutive enrolment of all-comers presenting in an ED setting of potentially critical ill patients.

Regarding risk for all-cause mortality, stroke and major bleedings, numerous clinical scores are currently used such as the CHA_2_DS_2_VASc-score, HASBLED, CHADS_2_, R_2_CHADS_2_, ATRIA, ORBIT or HEMORR_2_HAGES. Unfortunately, none of these scores has been validated for patients with impaired kidney function. Our findings demonstrate that the CHA_2_DS_2_VASc-score provides only a modest discrimination with an AUC: 0.624 (95%CI: 0.612–0.633) for prediction of stroke which is in line with previous studies [12, 24]. The previous evidence comes from real-world Euro Heart Survey for AF and from the RE-LY (Randomized Evaluation of Long-Term Anticoagulation Therapy) study reporting a modest performance with AUCs ranging between 0.61 to 0.56 for CHA_2_DS_2_VASc-score, CHADS_2_, biomarker-based ABC or ATRIA scores [12, 24]. Our findings now demonstrate that the addition of a reduced eGFR <60 ml/min at admission as a dichotomous variable did not improve the predictive information on risk of stroke but on risk for all-cause mortality and major bleedings.

When ORBIT and HASBLED score were compared concerning the assessment of bleeding risk, ORBIT score showed significantly better AUCs for prediction of major bleeding in all patients and even in patients with and without impaired kidney function compared to HASBLED score. These findings were confirmed using NRI with significantly better predictions of outcome events using ORBIT score compared to HASBLED score. Current evidence from a recent meta-analysis suggested that HASBLED and ORBIT score are of similar performance in assessing bleeding risk, supporting the use of HASBLED score in clinical practice [25, 26]. Hence, our real-world data for patients in an ED suggests a better performance of ORBIT compared to HASBLED score in predicting bleeding events in our non-high bleeding risk population of all-comer patients with AF.

In a meta-analysis including the RE-LY (Randomized Evaluation of Long Term Anticoagulant Therapy), ROCKET AF (The Rivaroxaban Once Daily Oral Direct Factor Xa Inhibition Compared with Vitamin K Antagonism for Prevention of Stroke and Embolism Trial in Atrial Fibrillation), ARISTOTLE (Apixaban for Reduction in Stroke and Other Thromboembolic Events in Atrial Fibrillation), and ENGAGE AF–TIMI 48 (Effective Anticoagulation with Factor Xa Next Generation in Atrial Fibrillation–Thrombolysis in Myocardial Infarction 48) trials [16] including a total of 71,683 AF patients who were randomized to receive DOAC or warfarin, DOACs were shown to significantly reduce the risk of stroke or systemic embolic events and also significantly reduced mortality compared to warfarin [16]. Herein, the authors assumed the reduced stroke risk for DOACs primary attributed to a reduction in hemorrhagic stroke events [16]. Additionally, there was no heterogeneity on the effect of DOAC versus warfarin for stroke or systemic embolic events in important subgroups, particularly with a consistent effect across renal function [16]. In agreement, patients in our registry treated with VKA showed a higher HR for all-cause mortality, major bleeding and MI but no significant excess for stroke compared to patients receiving DOAC regimes.

The generalizability of findings from RCTs to the real world is a subject of continued discussion [9], since there is a considerable variety in study designs, objectives and patient selection, as well as quality performance measures and standards [27, 28]. In some national registries, where data from an entire population is analysed, a selection bias could be assumed [29–31]. Finally, current RCTs and multicenter studies or registires substantially differ regarding their inclusion and exclusion criteria and therefore do not provide consecutive data reflecting the real-world [5, 8]. As such our findings are of particular interest as they reflect clinically relevant real-world evidence and add information on particular subsets of patients such as patients with reduced kidney function in the setting of an ED. Regarding the prevalence of moderate to severe CKD, the GARFIELD-AF registry (Global Anticoagulant Registry in the FIELD–Atrial Fibrillation) reported a prevalence of ~11% [8, 32]. In other registries the prevalence was found to range between 9.4% [33] to18.2% [34], and thus as frequent as in RCTs. In line with our findings, Laugesen et al. had conducted a nationwide survey on 1,560 patients with a prior diagnosis of AF and CKD enrolled from 2011–2017 in Denmark [2]. In this study DOAC use was associated with a significantly lower risk of major bleeding compared to VKA. An association between type of anticoagulant and risk of stroke was not reported. However, the investigators could not provide exact renal function for each patient and thus were not able to provide reliable data on the prognostic importance nor on the treatment effect of DOAC versus VKA across the spectrum of CKD. Our findings on the association between a reduced eGFR at presentation and adverse outcomes is consistent with results from major RCTs. Our findings also support previous observations that the CHA_2_DS_2_VASc-score not only predicts annual risk of stroke in AF but also incident all-cause mortality, MI and bleedings in AF patients and in a variety of cardiovascular diseases including CKD without AF [35], acute coronary artery syndrome [36] and heart failure [37] in the presence or absence of AF. In the Xantus (Xarelto for Prevention of Stroke in Patients with Atrial Fibrillation) registry low kidney function at baseline was the strongest predictor of a major bleeding event [33]. Regarding the beneficial effect of DOAC vs. VKA on the risk of major bleedings, our data are very consistent with findings from Laugesen et al. [2]. Moreover, our data suggest a benefit from DOAC vs. VKA for all-cause mortality and MI but similar to Laugesen et al. not for stroke.

However, there are a few limitations to consider. The limited number of study outcomes prevented us from analyzing differences among the individual types of DOAC. In addition, we could not provide data on appropriate dosing of non-vitamin K oral anticoagulants or acceptable therapeutic range ≥ 70% under VKA. Therefore, we cannot analyze outcomes of VKA or treatment effects with respect to time in TTR. Finally, we refrained from a sensitivity analysis on treatment benefits in patients with new initiation of DOAC versus VKA due to small numbers of patients and low event rates. For the same reason we did not investigate the treatment effect of individual DOAC regimes. In a meta-analysis of all randomized trials on treatment effects of patients randomized to DOACs or warfarin, low-dose DOAC regimens showed similar overall reductions in stroke or systemic embolic events compared to warfarin and a more favorable bleeding profile but significantly more ischemic strokes [16]. In the ENGAGE AF-TIMI 48 trial [38], comparisons were executed in the entire study population, irrespective whether they had received reduced doses of edoxaban at randomization due to an impaired kidney function CrCl≤50 ml/min. After excluding patients with an impaired kidney function defined by CrCl<30 mL/min and 30–50 mL/min at randomization a sensitivity analysis revealed comparable results for higher edoxaban dose regime compared to warfarin [38, 39]. Nevertheless, we cannot exclude that adjustments of our findings by appropriate dose reduction of DOAC or TTR for VKA could have an impact on our findings [39]. Additionally, there was no accounting for drug discontinuation, therefore bias attributing to discontinuations of OAC drugs could not be excluded. Despite the sequential follow-up within HERA-FIB, an underestimation of event rates owing the retrospective design of the study could not be fully excluded. Within HERA-FIB, bleeding events were classified according to ISTH major bleeding criteria, however this does not include data on the reason for the bleeding. Therefore, we could not provide a differentiation for bleeding reasons. Finally, as in most observational studies especially accounting for studies with patient requirement in an emergency department setting, unmeasured confounding or residual confounding affecting our results cannot be ruled out.

Taken together, the findings from our study confirmed data from RCTs concerning relevance of reduced kidney function in AF patients especially in the setting of an ED. Additionally, we could add evidence from a large single-center all-comer registry with AF patients for effectiveness and safety of DOAC regimes.

## Supporting information

S1 Fig Kaplan Meier analysis separated by eGFR stages for all-cause mortality (A), stroke (B), major bleeding events (C) and myocardial infarction (D). eGFR estimated GFR, MI, myocardial infarction.
(TIF)

S2 Fig Distribution of DOAC types per eGFR category of patients receiving OAC regimes within HERA-FIB. eGFR, estimated GFR.
(TIF)

S1 Table Individual components of CHA_2_DS_2_VASc, HAS BLED and ORBIT score. (DOCX)

S2 Table Cox regression model for all-cause mortality and univariate variables. (DOCX)

S3 Table Cox regression model for all-cause mortality and variables of CHA_2_DS_2_VASc score and presence of eGFR<60 ml/min. (DOCX)

S4 Table Cox regression model for ischemic stroke and variables of CHA_2_DS_2_VASc score and presence of eGFR<60 ml/min. (DOCX)

S5 Table Cox regression model for major bleedings and variables of CHA_2_DS_2_VASc score and presence of eGFR<60 ml/min. (DOCX)

S6 Table Cox regression model for myocardial infarction and variables of CHA_2_DS_2_VASc score and presence of eGFR<60 ml/min. (DOCX)

S7 Table Cox regression model for the composite endpoint and variables of CHA_2_DS_2_VASc score as well as eGFR<60 ml/min. (DOCX)

S8 Table Interaction analysis for the composite EP, significant univariate variables and severity of impaired kidney function per eGFR category. (DOCX)
