# Supplementary figures and images for: Effect of impaired kidney function on outcomes and treatment effects of oral anticoagulant regimes in patients with atrial fibrillation in a real-world registry
Source: PLoS One. 2024 Sep 23;19(9):e0310838. doi: 10.1371/journal.pone.0310838 (PMC11419350; doi:10.1371/journal.pone.0310838)

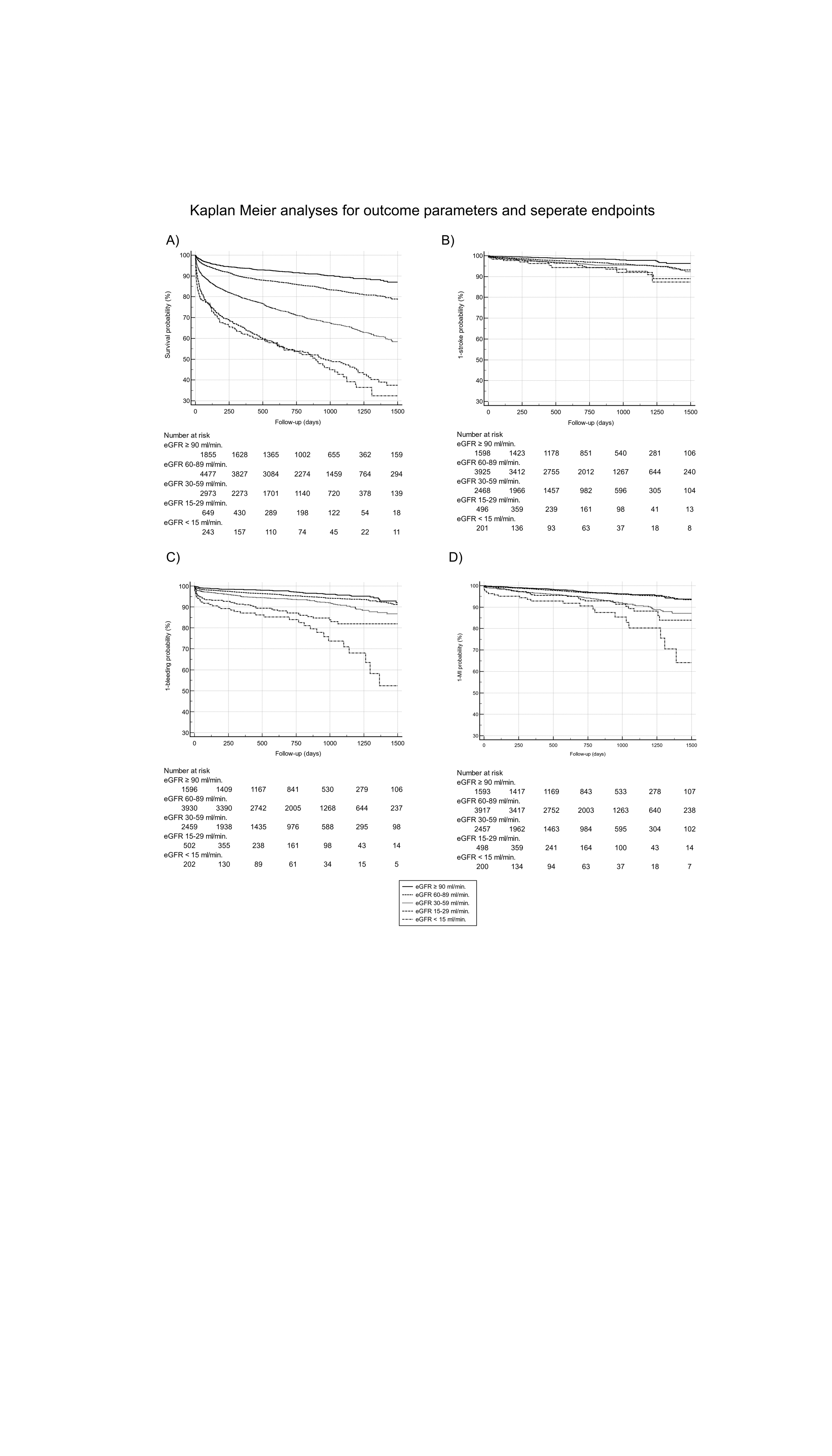

Supplement: S1 Fig — Kaplan Meier analysis separated by eGFR stages for all-cause mortality (A), stroke (B), major bleeding events (C) and myocardial infarction (D). eGFR estimated GFR, MI, myocardial infarction. (TIF) [file pone.0310838.s001.tif]

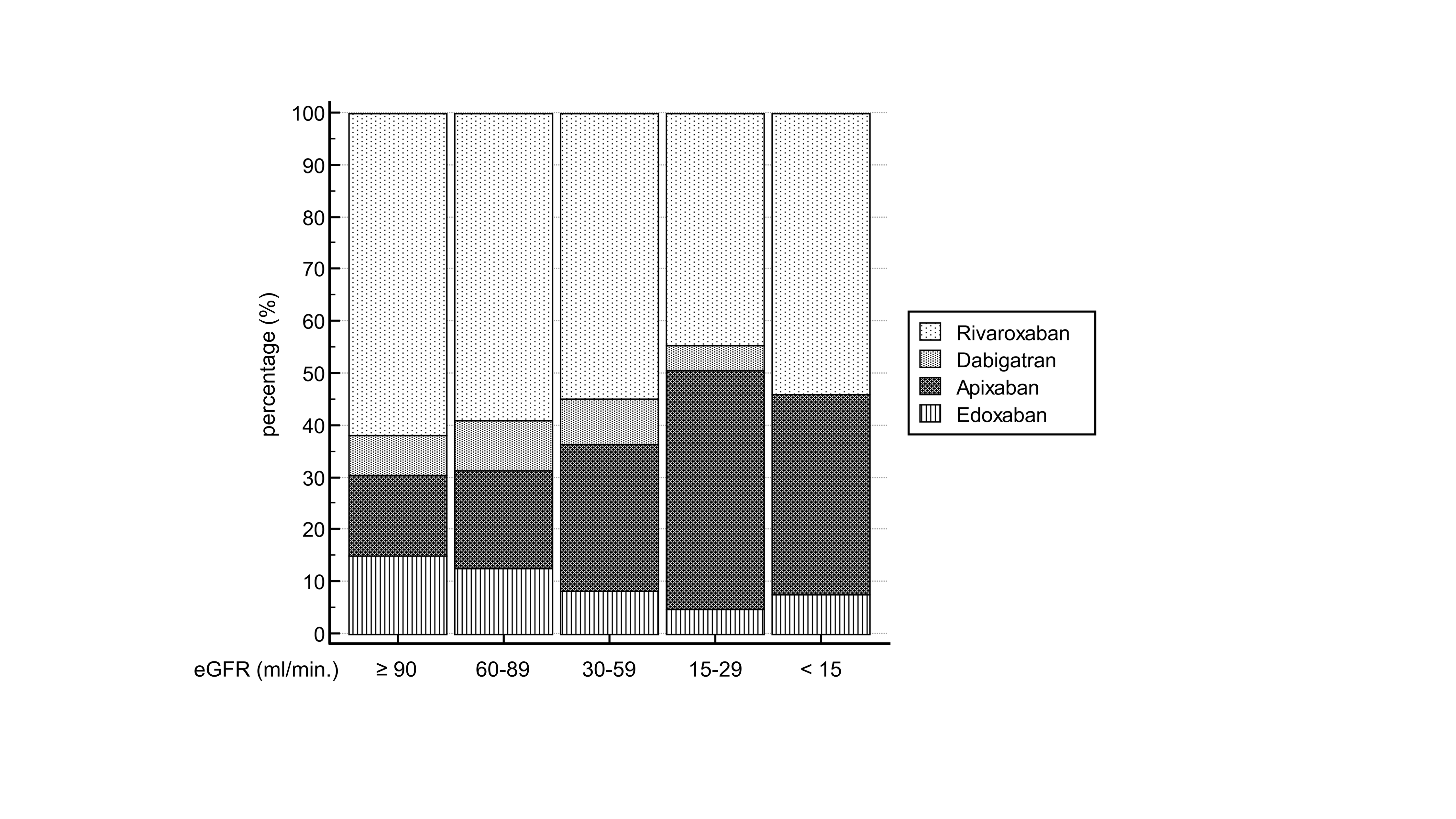

Supplement: S2 Fig — eGFR, estimated GFR. (TIF) [file pone.0310838.s002.tif]
