# Supplementary material for: Effect of impaired kidney function on outcomes and treatment effects of oral anticoagulant regimes in patients with atrial fibrillation in a real-world registry
Source: PLoS One. 2024 Sep 23;19(9):e0310838. doi: 10.1371/journal.pone.0310838 (PMC11419350; doi:10.1371/journal.pone.0310838)
Supplement: S1 Table — (DOCX) [file pone.0310838.s003.docx]

**S1 Table. Individual components of CHA_2_DS_2_VASc, HAS BLED and ORBIT score.**

| **Variables** | **eGFR ≥ 60 ml/min.** | **eGFR < 60 ml/min.** | **p-value** |
| --- | --- | --- | --- |
| Congestive heart failure, n (%_all_) | 1622 (25.6) | 1573 (40.4) | <0.001 |
| Arterial hypertension, n (%_all_) | 4913 (77.6) | 3523 (90.6) | <0.001 |
| Age ≥ 75 years, n (%_all_) | 2555 (40.3) | 2763 (71.1) | <0.001 |
| Age 65-74 years, n (%_all_) | 1785 (28.2) | 795 (20.4) | <0.001 |
| Diabetes mellitus, n (%_all_) | 1002 (15.8) | 1041 (26.8) | <0.001 |
| Stroke/TIA/thromboembolism, n (%_all_) | 658 (16.9) | 764 (12.1) | <0.001 |
| Vascular disease, n (%_all_) | 2624 (67.5) | 3090 (48.8) | <0.001 |
| Sex, female, n (%_all_) | 2465 (38.9) | 1800 (46.3) | <0.001 |
| Abnormal liver function, n (%_all_) | 42 (0.7) | 50 (1.3) | 0.001 |
| Abnormal kidney function, n (%_all_) | N/A | 606 (15.6) | <0.001 |
| History of severe bleeding, n (%_all_) | 245 (3.9) | 291 (7.5) | <0.001 |
| Labile INR, n (%_all_) | 38 (0.6) | 54 (1.4) | <0.001 |
| Age ≥ 65 years, n (%_all_) | 4340 (68.5) | 3558 (91.5) | <0.001 |
| Antiplatelet agents or NSAID, n (%_all_) | 1753 (27.7) | 1223 (31.5) | <0.001 |
| Alcohol use > 8u/week, n (%_all_) | 127 (2.0) | 69 (1.8) | 0.41 |
| Abnormal liver function, n (%_all_) | 42 (0.7) | 50 (1.3) | 0.001 |
| Abnormal kidney function, n (%_all_) | N/A | 606 (15.6) | <0.001 |
| Hb<13 g/dL (male)/Hb<12 g/dL (female), n (%_all_) | 1569 (24.8) | 2074 (53.3) | <0.001 |
| Prior treatment with antiplatelet agents, n (%_all_) | 1661 (26.2) | 1182 (30.4) | <0.001 |

TIA, transient ischemic attack; INR, international normalized ratio; NSAID, nonsteroidal anti-inflammatory drugs; Hb, hemoglobin.
