# Supplementary material for: Effect of impaired kidney function on outcomes and treatment effects of oral anticoagulant regimes in patients with atrial fibrillation in a real-world registry
Source: PLoS One. 2024 Sep 23;19(9):e0310838. doi: 10.1371/journal.pone.0310838 (PMC11419350; doi:10.1371/journal.pone.0310838)
Supplement: S2 Table — (DOCX) [file pone.0310838.s004.docx]

**S2 Table. Cox regression model for all-cause mortality and significant univariate variables.**

| **Covariate** | **aHR** | **95% CI** | **p-value** |
| --- | --- | --- | --- |
| Age | 1.05 | 1.04 – 1.05 | <0.0001 |
| Sex, male | 1.23 | 1.03 – 1.24 | 0.0087 |
| Arterial hypertension | 0.85 | 0.75 – 0.97 | 0.0148 |
| Diabetes mellitus | 1.13 | 1.03 – 1.25 | 0.0114 |
| Former CAD | 0.86 | 0.78 – 0.96 | 0.0069 |
| Former CABG | 1.16 | 1.01 – 1.34 | 0.0339 |
| Former myocardial infarction | 0.10 | 0.98 – 1.25 | 0.0977 |
| Former COPD | 1.43 | 1.28 – 1.60 | <0.0001 |
| Hsc-TnT ≥ 14 ng/L | 3.46 | 2.97 – 4.03 | <0.0001 |
| Oral anticoagulation | 0.37 | 0.34 – 0.40 | <0.0001 |
| eGFR 60-89 ml/min | 0.97 | 0.81-1.15 | 0.6985 |
| eGFR 30-59 ml/min | 1.35 | 1.13-1.62 | 0.0008 |
| eGFR 30-15 ml/min | 2.06 | 1.69-2.52 | <0.0001 |
| eGFR < 15 ml/min | 1.98 | 1.57-2.52 | <0.0001 |

aHR, adjusted hazard ratio; CAD, coronary artery disease; CABG, coronary artery bypass graft; COPD, chronic obstructive pulmonary disease; CI, confidence interval; eGFR, estimated GFR; hs-cTnT, high sensitive cardiac troponin T.
