# Supplementary material for: Effect of impaired kidney function on outcomes and treatment effects of oral anticoagulant regimes in patients with atrial fibrillation in a real-world registry
Source: PLoS One. 2024 Sep 23;19(9):e0310838. doi: 10.1371/journal.pone.0310838 (PMC11419350; doi:10.1371/journal.pone.0310838)
Supplement: S3 Table — (DOCX) [file pone.0310838.s005.docx]

**S3 Table. Cox regression model for all-cause mortality and variables of CHA_2_DS_2_VASc-score** **and presence of eGFR<60 ml/min.**

| **Covariate** | **aHR** | **95% CI** | **p-value** |
| --- | --- | --- | --- |
| Congestive heart failure | 1.23 | 1.13 – 1.34 | <0.0001 |
| Arterial Hypertension | 0.85 | 0.74 – 0.97 | 0.0113 |
| Age ≥ 75 years | 3.12 | 2.65 – 3.67 | <0.0001 |
| Age 65 - 75 years | 1.46 | 1.22 – 1.75 | <0.0001 |
| Diabetes mellitus | 1.31 | 1.19 – 1.45 | <0.0001 |
| Former TIA/stroke/thromboembolism | 1.20 | 1.07 – 1.33 | 0.0016 |
| Former vascular disease | 1.03 | 0.94 – 1.12 | 0.5824 |
| Gender (female) | 0.61 | 0.27 – 1,35 | 0.2197 |
| eGFR< 60 ml/min. | 2.18 | 2.00 – 2.40 | <0.0001 |

aHR, adjusted hazard ratio; CI, confidence interval; TIA, transient ischemic attack; eGFR, estimated GFR.
