# Supplementary material for: Effect of impaired kidney function on outcomes and treatment effects of oral anticoagulant regimes in patients with atrial fibrillation in a real-world registry
Source: PLoS One. 2024 Sep 23;19(9):e0310838. doi: 10.1371/journal.pone.0310838 (PMC11419350; doi:10.1371/journal.pone.0310838)
Supplement: S4 Table — (DOCX) [file pone.0310838.s006.docx]

**S4 Table. Cox regression model for ischemic stroke and variables of CHA_2_DS_2_VASc-score** **and presence of eGFR<60 ml/min.**

| **Covariate** | **aHR** | **95% CI** | **p-value** |
| --- | --- | --- | --- |
| Congestive heart failure | 1.16 | 0.91 – 1.50 | 0.2305 |
| Arterial Hypertension | 1.47 | 0.97 – 2.23 | 0.0719 |
| Age ≥ 75 years | 3.23 | 2.07 – 5.02 | <0.0001 |
| Age 65 - 75 years | 2.23 | 1.40 – 3.54 | 0.0007 |
| Diabetes mellitus | 1.78 | 1.38 – 2.30 | <0.0001 |
| Former TIA/stroke/ thromboembolism | 1.50 | 1.12 – 2.01 | 0.0066 |
| Former vascular disease | 0.96 | 0.76 – 1.22 | 0.7558 |
| Gender (female) | 0.84 | 0.12 – 6.03 | 0.8653 |
| eGFR< 60 ml/min. | 1.08 | 0.84 – 1.38 | 0.5500 |

aHR, adjusted hazard ratio; CI, confidence interval; TIA, transient ischemic attack; eGFR, estimated GFR.
