# Supplementary material for: Effect of impaired kidney function on outcomes and treatment effects of oral anticoagulant regimes in patients with atrial fibrillation in a real-world registry
Source: PLoS One. 2024 Sep 23;19(9):e0310838. doi: 10.1371/journal.pone.0310838 (PMC11419350; doi:10.1371/journal.pone.0310838)
Supplement: S5 Table — (DOCX) [file pone.0310838.s007.docx]

**S5 Table. Cox regression model for major bleedings and variables of CHA_2_DS_2_VASc-score** **and presence of eGFR<60 ml/min.**

| **Covariate** | **aHR** | **95% CI** | **p-value** |
| --- | --- | --- | --- |
| Congestive heart failure | 1.50 | 1.26 - 1.80 | <0.0001 |
| Arterial Hypertension | 1.74 | 1.27 - 2.39 | 0.0006 |
| Age ≥ 75 years | 1.39 | 1.07 - 1.81 | 0.0156 |
| Age 65 - 75 years | 1.03 | 0.77 - 1.34 | 0.8649 |
| Diabetes mellitus | 1.22 | 0.99 - 1.49 | 0.0631 |
| Former TIA/stroke/thromboembolism | 1.23 | 0.97 - 1.55 | 0.0820 |
| Former vascular disease | 0.96 | 0.80 - 1.15 | 0.6306 |
| Gender (female) | 0.98 | 0.24 - 3.93 | 0.9762 |
| eGFR< 60 ml/min. | 1.68 | 1.40 - 2.03 | <0.0001 |

aHR, adjusted hazard ratio; CI, confidence interval; TIA, transient ischemic attack; eGFR, estimated GFR.
