# Supplementary material for: Effect of impaired kidney function on outcomes and treatment effects of oral anticoagulant regimes in patients with atrial fibrillation in a real-world registry
Source: PLoS One. 2024 Sep 23;19(9):e0310838. doi: 10.1371/journal.pone.0310838 (PMC11419350; doi:10.1371/journal.pone.0310838)
Supplement: S6 Table — (DOCX) [file pone.0310838.s008.docx]

**S6 Table. Cox regression model for myocardial infarction and variables of CHA_2_DS_2_VASc-score** **and presence of eGFR<60 ml/min.**

| **Covariate** | **aHR** | **95% CI** | **p-value** |
| --- | --- | --- | --- |
| Congestive heart failure | 1.31 | 1.06 - 1.61 | 0.0113 |
| Arterial Hypertension | 1.15 | 0.81 - 1.63 | 0.4386 |
| Age ≥ 75 years | 1.94 | 1.38 - 2.73 | 0.0002 |
| Age 65 - 75 years | 1.35 | 0.94 - 1.94 | 0.1079 |
| Diabetes mellitus | 1.76 | 1.41 - 2.18 | <0.0001 |
| Former TIA/stroke/thromboembolism | 1.02 | 0.77 - 1.34 | 0.9048 |
| Former vascular disease | 2.03 | 1.64 - 2.53 | <0.0001 |
| Gender (female) | 0.53 | 0.08 - 3.80 | 0.5306 |
| eGFR< 60 ml/min. | 1.59 | 1.29 - 1.97 | <0.0001 |

aHR, adjusted hazard ratio; CI, confidence interval; TIA, transient ischemic attack; eGFR, estimated GFR.
