# Supplementary material for: Effect of impaired kidney function on outcomes and treatment effects of oral anticoagulant regimes in patients with atrial fibrillation in a real-world registry
Source: PLoS One. 2024 Sep 23;19(9):e0310838. doi: 10.1371/journal.pone.0310838 (PMC11419350; doi:10.1371/journal.pone.0310838)
Supplement: S7 Table — (DOCX) [file pone.0310838.s009.docx]

**S7 Table. Cox regression model for the composite endpoint and variables of CHA_2_DS_2_VASc-score as well as eGFR<60 ml/min.**

| **Covariate** | **aHR** | **95% CI** | **p-value** |
| --- | --- | --- | --- |
| Congestive heart failure | 1.23 | 1.14 - 1.33 | <0.0001 |
| Arterial Hypertension | 0.98 | 0.87 - 1.10 | 0.7361 |
| Age ≥ 75 years | 2.54 | 2.23 - 2.90 | <0.0001 |
| Age 65 - 75 years | 1.41 | 1.22 - 1.63 | <0.0001 |
| Diabetes mellitus | 1.36 | 1.25 - 1.48 | <0.0001 |
| Former TIA/stroke/thromboembolism | 1.21 | 1.10 - 1.33 | 0.0001 |
| Former vascular disease | 1.10 | 0.99 – 1.15 | 0.0958 |
| Gender (female) | 0.68 | 0.36 - 1.32 | 0.2543 |
| eGFR< 60 ml/min. | 1.85 | 1.71 – 2.00 | <0.0001 |

aHR, adjusted hazard ratio; CI, confidence interval; TIA, transient ischemic attack; eGFR, estimated GFR.
