# Supplementary material for: Effect of impaired kidney function on outcomes and treatment effects of oral anticoagulant regimes in patients with atrial fibrillation in a real-world registry
Source: PLoS One. 2024 Sep 23;19(9):e0310838. doi: 10.1371/journal.pone.0310838 (PMC11419350; doi:10.1371/journal.pone.0310838)
Supplement: S8 Table — (DOCX) [file pone.0310838.s010.docx]

**S8 Table. Interaction analysis for the composite EP, significant univariate variables and severity of impaired kidney function per eGFR category.**

| **variables** | **aHR** | **95%CI** | **p-value** | **P interaction*** |
| --- | --- | --- | --- | --- |
| Age | 1.03 | 1.02 - 1.03 | <0.001 | <0.001 |
| Sex, male | 0.90 | 0.83 - 0.98 | 0.0151 | 0.3544 |
| Arterial hypertension | 0.96 | 0.85 - 1.08 | 0.5264 | 0.1577 |
| Diabetes mellitus | 1.37 | 1.07 - 1.76 | 0.0114 | 0.2371 |
| Former CAD | 0.93 | 0.84 - 1.02 | 0.1200 | 0.8382 |
| Former CABG | 1.41 | 0.99 - 1.99 | 0.0534 | 0.3605 |
| Former MI | 1.10 | 0.99 - 1.23 | 0.0727 | 0.4423 |
| Former COPD | 1.80 | 1.34 - 2.41 | 0.0001 | 0.0222 |
| Hsc-TnT ≥ 14 ng/L | 2.71 | 2.39 - 3.08 | <0.0001 | 0.1214 |
| No oral anticoagulation | 2.27 | 2.08 - 2.47 | <0.0001 | 0.8873 |

*p-value of interaction was calculated for eGFR stages and respective variables. CAD, coronary artery disease; CABG, coronary artery bypass grafting, MI, myocardial infarction, COPD, chronic obstructive pulmonary disease, hsc-TnT, high sensitive cardiac troponin T.
